# Patterns of care for patients with advanced soft tissue sarcoma: experience from Australian sarcoma services

**DOI:** 10.1186/s13569-016-0052-4

**Published:** 2016-07-11

**Authors:** Susie Bae, Philip Crowe, Raghu Gowda, Warren Joubert, Richard Carey-Smith, Paul Stalley, Jayesh Desai

**Affiliations:** Department of Medical Oncology, Peter MacCallum Cancer Centre, St Andrews Place, East Melbourne, VIC 3002 Australia; Department of Surgery, Prince of Wales Hospital, Barker St, Randwick, NSW 2031 Australia; Department of Radiation Oncology, Royal Adelaide Hospital, North Terrace, Adelaide, SA 5000 Australia; Department of Medical Oncology, Princess Alexandra Hospital, 199 Ipswich Rd, Woolloongabba, QLD 4102 Australia; Department of Orthopaedic Surgery, Sir Charles Gardner Hospital, Hospital Ave, Nedlands, WA 6009 Australia; Department of Orthopaedic Surgery, Royal Prince Alfred Hospital, Missenden Rd, Camperdown, NSW 2050 Australia

**Keywords:** Advanced soft tissue sarcoma, Metastatic sarcoma, Palliative-intent treatment, Patterns of care study, Chemotherapy, Radiotherapy, Metastasectomy

## Abstract

**Background:**

There is a paucity of data on the current management of patients with advanced soft tissue sarcoma (STS) in the Australian health care setting. This study utilised the Australian sarcoma database to evaluate the patterns of care delivered to patients with advanced STS at Australian sarcoma services.

**Methods:**

Prospectively collected data from six sarcoma centres in Australia were sourced to identify patients diagnosed with advanced STS between 1 January 2010 and 31 December 2012. Descriptive statistics were analysed for patient demographics, clinicopathological characteristics and treatment patterns. Overall survival was estimated using the Kaplan–Meier product limit method.

**Results:**

Of 253 patients with advanced STS, four major STS subtypes were identified: undifferentiated pleomorphic sarcoma (23 %), leiomyosarcoma (17 %), liposarcoma (14 %), and synovial sarcoma (8 %); with the rest grouped as “other STS” (38 %). Approximately one-third of patients received palliative systemic therapy with the most common first-line therapy being doxorubicin alone (50 %). A small percentage of patients participated in clinical trials (20 %). Palliative radiotherapy was utilised mostly for treatment of symptomatic distant metastases and one-third of patients underwent metastasectomy, most commonly for pulmonary metastases. The median overall survival (OS) in this series was 18 months and no significant difference in OS was observed across different STS histological subtypes.

**Conclusions:**

This is the first detailed study outlining patterns of care for Australian patients with advanced STS managed at sarcoma services. These data highlight a particular area of weakness in the lack of clinical trials for sarcoma patients and also serve as an important reference point for understanding how practice may change over time as treatment options evolve.

## Background

Soft tissue sarcomas (STS) comprise a heterogeneous group of diseases, accounting for less than 1 % of adult malignancies [1]. Metastatic or locally advanced STS is generally considered incurable with the mainstay of treatment being systemic chemotherapy. For some patients with limited disease burden however, long-term remission can be achieved through a multimodality approach involving medical, surgical and radiation therapy. The goal of treatment here is to prolong survival whilst maintaining or improving quality of life and dealing with specific disease-related symptoms.

In clinical practice, the decision-making process regarding the choice of systemic therapy, the timing of treatment and the use of single versus combination therapy is highly complex. International guidelines such as the National Comprehensive Cancer Network and the European Society of Medical Oncology offer some consensus on the first-line treatment with anthracycline-based therapy [2, 3]. However, systemic therapy options remain limited beyond the first- and second-line therapy with a lack of evidence for improvement in overall survival with anthracycline-containing doublet chemotherapy [4–7]. Newer agents such as trabectedin and pazopanib with promising activity for certain histologic subtypes have been identified, but these remain a minority and access continues to be a challenge for the treating team and their patients. As novel agents are developed and approved by the regulatory authority, access is highly variable across geographical regions around the globe. Ostensibly these differences in access to effective drugs influence treatment choice but little is known about how treatment algorithms are modified accordingly in routine clinical practice.

A recent study by the North American and European colleagues described chemotherapy treatment patterns and clinical outcomes for patients with metastatic STS highlighting the poor overall survival for this group and the need for more therapeutic options [8]. There is a paucity of data on the current practice of managing patients with advanced STS in the Australian health care setting. The primary goal of this study was to utilise the Australian sarcoma database to evaluate the current patterns of care for patients with advanced STS, managed at sarcoma specialist centres.

## Methods

A customised electronic database capturing clinical data considered most relevant to bone and soft tissue sarcomas were established at major sarcoma centres around Australia, with data collection commencing in 2009, approved by the governing ethics committee at each institution. Data from six sarcoma centres were sourced to identify patients diagnosed with advanced STS between 1st January 2010 and 31st December 2012, with a minimum follow up period of 12 months. Subjects aged 18 and above with locally advanced and/or metastatic STS were selected for inclusion into the study if they received care at sarcoma specialist centres with at least two visits during the study period. Data on all systemic therapies given with palliative-intent were collated. Information on palliative therapy other than systemic therapy, i.e. radiotherapy and/or surgery, was available in 89 % of the total study population (n = 225) for analysis. The BioGrid Australia platform was utilised to link the datasets for analysis. The study was approved by the Human Research Ethics Committee at Melbourne Health.

### Definitions

- Advanced STS

We defined patients with advanced STS as patients with metastatic and/or locally advanced, unresectable histologically confirmed STS demonstrated by appropriate imaging and biopsy. For the purpose of this study, we excluded patients with gastrointestinal stromal tumour, bone sarcomas, dermatofibrosarcoma protuberans, or rhabdomyosarcomas. (Table 1)

**Table 1:** Soft tissue sarcoma WHO classification subtypes included and excluded in the study

Included histological subtypes	Excluded histological subtypes
Fibroblastic sarcoma Undifferentiated pleomorphic sarcoma Leiomyosarcoma Adipocytic sarcoma Vascular sarcoma Sarcomas of uncertain differentiation; including synovial sarcoma, malignant peripheral nerve sheath tumour, undifferentiated soft tissue sarcoma not otherwise specified	Rhabdomyosarcoma Chondrosarcoma Osteosarcoma Ewing family tumour Gastro-intestinal stromal tumour Dermatofibromatosis sarcoma protuberans Inflammatory myofibroblastic sarcoma Malignant mesothelioma Mixed mesodermal tumours of the uterus Kaposi’s sarcoma Desmoid Giant cell tumour

- Palliative treatment modalities

We defined systemic chemotherapy, radiotherapy and surgery as palliative treatment modalities when they were used for the purpose of disease and symptom control in patients with advanced STS.

### Statistical methods

Descriptive statistics were analysed for patients’ demographics, clinicopathological characteristics and treatment patterns. Overall survival (OS) was estimated for the whole patient cohort using the Kaplan–Meier product limit method and separately for different STS subgroups. The prognostic impact of these variables was explored via the log-rank test. Statistical calculations were performed using SAS Enterprise Guide 6.1 (SAS institute Inc, Cary, NC, USA).

## Results

A total of 942 individuals were identified as having histologically confirmed STS diagnosed between 1^st^ January 2010 and 31st December 2012, of which 253 (27 %) were considered as having advanced STS. Of this group, 34 % (n = 86) had metastatic disease at diagnosis, with the remainder presenting with local recurrence that was unresectable and/or distant metastatic disease. The mean age at diagnosis was 59 years (range; 18–95). There was a slight male preponderance with 149 males and 104 females represented in the cohort. The most frequent primary tumour sites were extremity (45 %), followed by retroperitoneum (15 %), intra-abdominal and intra-thoracic (11 % each). The lung was the most common site of distant metastasis (66 %), followed by intra-abdomen (28 %) and bone (19 %).

Based on tumour histology, the study cohort (n = 253) was classified into four major STS subtypes: undifferentiated pleomorphic sarcoma (n = 57; 23 %), leiomyosarcoma (n = 44; 17 %), liposarcoma (n = 34; 14 %), synovial sarcoma (n = 21; 8 %); with the rest grouped as “other STS” (n = 97; 38 %); consisting of 15 histologic subtypes, each with small sample sizes. The uptake of palliative-intent systemic therapy differed across the STS histological subtypes with patients with synovial sarcomas most likely to be treated with systemic therapy compared to other subtypes (Table 2).

**Table 2:** Demographics and clinical characteristics of patients with advanced STS

Characteristic	Total (n = 253)	Treated group^a^ (n = 86)	Not treated group^b^ (n = 167)	*P*
*Age at diagnosis*				
Mean (range)	59 (18–91)	51 (18–85)	63 (22–95)	N/A
Number of patients				
<65 years, n (%)	147 (58)	68 (27)	79 (31)	0.0001
≥65 years, n (%)	106 (42)	18 (7)	88 (35)	
*Gender, n (%)*				
Female	104 (41)	37 (15)	67 (26)	0.687
Male	149 (59)	49 (19)	100 (40)	
Stage IV at diagnosis	86 (34)	32 (13)	51 (20)	0.323
n (%)				
*Histological type, n (%)*				
UPS^c^	57 (23)	21 (8)	36 (14)	N/A
Leiomyosarcoma	44 (17)	22 (9)	22 (9)	
Liposarcoma	34 (14)	9 (4)	25 (10)	
Synovial sarcoma	21 (8)	15 (6)	6 (2)	
Other	97 (38)	19 (7)	78 (31)	
*Sites of metastases, n (%)* ^*d*^				
Lung	167 (66)	78 (31)	89 (35)	N/A
Intra-abdominal	70 (28)	32 (13)	38 (15)	
Bone	48 (19)	28 (11)	20 (8)	
Lymph node	44 (17)	16 (6)	18 (7)	
Head and neck	6 (2)	3 (1)	3 (1)	
Other	27 (11)	10 (4)	17 (7)	

^a^Group treated with palliative-intent systemic therapy

^b^Group not treated with palliative-intent systemic therapy

^c^UPS; undifferentiated pleomorphic sarcoma

^d^This value dose not add up to 100 % due to some having multiple sites of distant disease at different time points

### Palliative treatment modalities

Palliative treatment other than conventional cytotoxic chemotherapy was reviewed in those with available data (n = 225). A total of 86 patients (34 %) were treated with systemic therapies for advanced STS. More than a third of patients underwent radiotherapy or metastasectomy for palliation (37 %; radiotherapy, 35 %; surgery).

Approximately 75 % of all patients diagnosed with advanced STS received at least one line of palliative-intent treatment modality. Fifty-five patients (22 %) underwent two or more lines of different modalities of treatment during their course of disease. There were 11 patients (4 %) who received all three treatment modalities. Those who were treated with systemic therapies tended to be younger (mean age 51 vs. 63 years) and were more likely to receive radiotherapy for palliation than those who were not treated with systemic therapies (49 vs. 33 % respectively). There was a similar male to female ratio in each group.

### Systemic treatment patterns

Of the 86 patients who received palliative-intent systemic therapy, 50 % (n = 43) received more than one line of systemic therapy (Table 3). In the first-line setting, the most common regimen used was doxorubicin alone (n = 43, 55 %) followed by combination therapy with doxorubicin (or epirubicin in one patient) and ifosfamide (n = 14, 16 %). The majority of patients (n = 62, 72 %) received single-agent chemotherapy as the first-line therapy. Patients receiving combination chemotherapy were younger than those receiving monotherapy (mean age, 46 vs. 53 years respectively); with synovial sarcoma the most common subtype to receive doublet chemotherapy with doxorubicin and ifosfamide (n = 5, 38 %). Other doublet regimens used as the first-line therapy included the combination of docetaxel and gemcitabine (n = 2), and that of gemcitabine and dacarbazine (n = 2). Fourteen patients (16 %) were enrolled in clinical trials for the first-line therapy. In the second-line setting, ifosfamide was the most frequently prescribed agent (n = 20, 53 %) with the majority of patients receiving it as monotherapy (n = 35, 92 %) and with three additional patients participating in clinical trials. In the third-line setting, various regimens of monotherapy and combination therapies were prescribed with dacarbazine, most commonly delivered as a single-agent (n = 7, 30 %). Newer systemic agents such as multi-targeted receptor tyrosine kinase inhibitor, pazopanib and marine alkaloid, trabectedin, were sporadically used as subsequent lines of therapy (pazopanib; n = 12, trabectedin; n = 3). Beyond the first-line treatment, the most commonly used combination regimen was gemcitabine and docetaxel. Eight patients were treated with oral cyclophosphamide at any given time period (first-line; n = 3, second-line; n = 1, third-line; n = 3, fourth-line; n = 1). No patients were enrolled in clinical trials beyond the second-line setting. Prior exposure to neoadjuvant or adjuvant chemotherapy (n = 41, 16 %) and STS histological subtype did not affect the subsequent use of systemic therapy for advanced disease.

**Table 3:** Summary of palliative systemic therapy in treated patients

Systemic therapy line, n (%^a^)	Treatment details
First-line (n = 86, 100 %)	Single-agent chemtoherapy (n = 62, 72 %)
	Doxorubicin (n = 43)
	Clinical trial (n = 7)
	Ifosfamide (n = 4)
	Paclitaxel (n = 3)
	Other (n = 5)
	Combination therapy (n = 24, 28 %)
	Doxorubicin^b^ and ifosfamide (n = 13)
	Clinical trial (n = 7)
	Other combination (n = 4)
Second-line (n = 38, 44 %)	Single-agent chemtoherapy (n = 35, 92 %)
	Ifosfamide (n = 20)
	Dacarbazine (n = 5)
	Pazopanib (n = 3)
	Clinical trial (n = 3)
	Other (n = 4)
	Combination therapy (n = 3, 8 %)
Third-line (n = 20, 23 %)	Single-agent chemtoherapy (n = 18, 90 %)
	Pazopanib (n = 6)
	Dacarbazine (n = 6)
	Cyclophophamide (n = 3)
	Other (n = 3)
	Combination therapy (n = 2, 10 %)
Fourth-line (n = 6, 7 %)	Single-agent chemtoherapy (n = 6, 100 %)
	Pazopanib (n = 2)
	Doxorubicin (n = 1)
	Ifosfamide (n = 1)
	Cyclophophamide (n = 1)
	Trabectedin (n = 1)
Fifth-line (n = 1, 1 %)	Pazopanib (n = 1)

^a^The percentage refers to the proportion of patients who received the corresponding line treatment out of the total number treated with systemic therapy (n = 86)

^b^One patient had epirubicin and ifosfamide

### Other treatment patterns

Details of palliative-intent radiotherapy delivery and metastasectomy were available in 89 % of patients in this study (n = 225). The majority of patients received palliative-intent radiotherapy for symptoms arising from distant metastases (n = 81, 36 %), of which the most common sites included bone, followed by lung/mediastinum and intra-abdominal metastases. A further 34 % of patients with advanced STS underwent resection of metastases, of which pulmonary metastasectomy was the most common procedure performed. Approximately 10 % of patients underwent more than one metastasectomies.

### Survival analysis

**Fig. 1:**
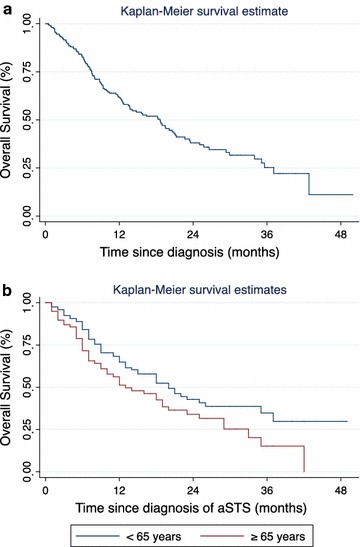
**a** Kaplan–Meier curves for overall survival. Overall survival for the whole patient cohort. **b** Kaplan–Meier curves for overall survivsl. Overall survival stratified by age group, <65 years versus ≥65 years

There were 110 deaths captured in this series, accounting for 43 % of the patients with advanced STS in this study cohort. The median overall survival in this series was 18 months (Fig. 1). Younger age at diagnosis of advanced STS (<65 years) conferred a statistically significant higher probability of survival than older age (≥65 years; log rank test *p* value 0.032). There was no statistically significant difference in overall survival stratified by STS histological subtypes. In the subgroup of patients who had one or more lines of systemic therapy, the median overall survival was 11 months from the time of diagnosis with advanced STS.

## Discussion

To our knowledge, this is the first detailed study outlining patterns of care for patients with advanced STS in Australian sarcoma centres using a prospectively maintained sarcoma-specific database. The STS histological subtypes represented in this series were comparable to other studies on patients with STS [8, 9]. Although each subtype was small in number, the study findings provide valuable insights into how patients with advanced STS are routinely managed in real-world clinical practice. Consistent with the literature, the most common site of distant metastasis was lung, and this was also reflected by a high uptake of pulmonary metastasectomy.

Over three quarters of all patients diagnosed with advanced STS received at least one modality of palliative-intent treatment. Systemic treatment, surgery and radiotherapy were used with almost equal distribution during the course of disease. A higher proportion of patients treated with systemic therapy also received palliative-intent radiotherapy than those who did not receive systemic therapy (49 vs. 33 %). The process involved in treatment decision-making is often complex and there may be several reasons for not recommending treatment for some patients with advanced STS. This may reflect the natural history of STS, which may exhibit more indolent course of disease with a lack of or minimal symptoms requiring intervention. Soft tissue sarcomas are generally considered less responsive to systemic chemotherapy than bone sarcomas such as osteosarcoma or Ewing’s sarcoma. As a result, depending on the overall disease burden, it may be appropriate to delay starting chemotherapy until it is required. Patients’ performance status and comorbidities should also be weighed against potential treatment toxicities. Little is known about how these variables influence the treatment decision and the patients’ overall outcome. It would be valuable to explore these further in subsequent studies.

Therapeutic options for many solid tumours have rapidly expanded and evolved over the last decades, however, patients with STS continue to experience difficulties with limited therapeutic options. Traditionally, doxorubicin-based regimens have been the standard of care [4–7, 10] and this was similarly observed in our practice with doxorubicin monotherapy or doxorubicin-containing combination chemotherapy as the most frequently prescribed first-line systemic therapy (n = 56, 65 %). In cases where other agents were administered, this was largely due to enrolment in clinical trials or the use of subtype-specific agents such as paclitaxel in angiosarcomas or gemcitabine and docetaxel in uterine leiomyosarcomas [11–13]. Interestingly, the uptake of doublet chemotherapy with gemcitabine and docetaxel was low at 7 % in Australia. This compares to approximately 30 % reported from the SABINE study and another single-institution North American study [8, 9]. This discrepancy may be related to differences in drug reimbursement available at the time of the study and a subsequent follow-up study will be of value to further characterise chemotherapy prescribing patterns impacted by changes in reimbursement over time. The uptake of doublet chemotherapy was also commonly seen, mostly in the younger patient groups with locally advanced STS. Given the evidence supporting the use of doxorubicin-based combination chemotherapy in improving the response rate at the expense of increased toxicities, this seems appropriate [2, 4–7].

It is well recognised that certain histological subtypes may have higher rates of chemo-sensitivity which may factor into the decision-making process for timing and type of treatment. Examples include ifosfamide with synovial sarcoma, taxanes with angiosarcoma and trabectedin with myxoid/round cell liposarcomas [11, 12, 14–16]. A closer review of treatment according to STS histological subtypes revealed a trend for an increased use of systemic therapy in patients with leiomyosarcoma and synovial sarcoma compared to the rest of histological subtypes including those with liposarcoma and undifferentiated pleomorphic sarcoma (Table 2). However, the small sample size of each histological subtype was insufficient to allow conclusive results.

Another salient finding from this study was the poor participation rate of patients with advanced STS in clinical trials. Approximately 20 % of patients treated with chemotherapy were enrolled in clinical trials during their course of disease with the majority participating in Phase III studies as the first-line therapy. This represents a strikingly small number of participants in contrast to 56–67 % of patients accessing clinical trials in European and North American settings [8, 9]. Australian medical oncologists are actively engaged in local and international cancer clinical trials. However, the heterogeneity and the rarity of soft tissue sarcoma, combined with the relative geographical isolation of Australia pose significant challenges in developing and opening clinical trials. Enhancing clinical trial access is critical, not only to improve cancer outcomes, but also to empower patients to play an active role in their management and to gain access to new treatments before they become widely available. Recent efforts by the Australian Sarcoma Study Group have facilitated multiple Australian and New Zealand sites to participate in investigator-initiated research as well as internationally-led cooperative group trials [17]. This is an important step forward in fostering relationship with future partners for sarcoma initiatives and research projects.

Access to new emerging agents continues to be a barrier to Australian patients. A total of 15 patients accessed novel agents, pazopanib (n = 12) and trabectedin (n = 3), for various STS subtypes, via compassionate access schemes. Although benefit in progression-free survival was not assessed in this series, these agents serve as additional active agents, which are important for patients with advanced STS. In general, timelines for access to new agents in Australia is lagging behind that of Europe and the United States. It is worth noting that it took seven more years for trabectedin to obtain a seal of approval by the Food and Drug Administration of the United States since its first approval in Europe in 2007. Trabectedin is yet to be approved by the Australian regulatory authority. On the other hand, pazopanib, has been added to the Pharmaceutical Benefits Scheme listing in March 2014 in Australia for the indication of non-adipocytic STS and will assist patients’ access to this targeted option at a Government-subsidised price [18].

The median overall survival of patients in our cohort was 18 months, which is longer than previous studies reporting approximately 12 months in this setting [19, 20]. However, long-term survival has been described in a subset of patients with limited disease burden and certain STS histologic subtypes are well recognised for their slow and indolent natural history. It is worth noting that the SABINE study evaluating a highly selected group of patients with a favourable response to chemotherapy reported the median overall survival as 23 months [8]. Interestingly, the group of patients who received palliative-intent systemic therapy in our study had the overall survival of only 11 months, which is more consistent with the historical control [19, 20]. This may represent the clinician’s decision in selecting patients with symptomatic and larger disease burden to receive systemic therapy. Not surprisingly in the setting of advanced or metastatic disease, the cause of death in most patients was disease-related.

There are several strengths to our data including the prospective nature of comprehensive data collection from the time of diagnosis through to recurrence and death. Details on clinic-pathological data as well as treatments were complete for analysis in most patients. Given the intrinsic heterogeneity of STS and its rarity, a considerable level of complexity exists in capturing an accurate dataset. However, a high quality, well-maintained database can be utilised as an important resource for research, including many questions not adequately addressed by clinical trials. Some limitations are worth noting. Data on multiple STS histological subtypes were combined together for this analysis, as they are commonly done in other studies. This renders interpretation of results difficult in treatment uptake and survival, as certain histological subtypes are inherently different from one another.

## Conclusions

In this retrospective study, we reviewed the patterns of care in managing patients with locally advanced and/or metastatic STS at sarcoma specialist services, providing a valuable insight into the current practice in the Australian health care setting. The presented data highlight varying practice in delivering palliative-intent treatment modalities at different time points and the ongoing need for addressing lack of clinical trials for sarcoma patients in Australia. The study will serve as an important reference point for understanding how practice may change over time as treatment options continue to evolve.

## Abbreviations

PFS: progression-free survival
OS: overall survival
STS: soft tissue sarcoma
